# Conflict of interest and professional ethics

**DOI:** 10.1186/1824-7288-41-S2-A65

**Published:** 2015-09-30

**Authors:** Stefano Semplici

**Affiliations:** 1 University of Rome “Tor Vergata”, Rome, 00173, Italy

## 

In general terms, we can face a conflict of interest every time that we have to deal with competing goals and responsibilities. In a more specific sense, however, such a conflict is understood as a situation where a personal self-interest could unduly interfere with professional responsibilities. This situation appears particularly blameworthy, when public interest and protection of fundamental individual rights are involved. According to the Italian Code of medical deontology, conflict of interest is in itself a *condition* that a physician may well happen to experience and not *ipso facto* a violation of legal or moral rules. As a condition, it entails the necessity to make decisions which should always comply with an unquestionable priority: the best interest of the patient must always come first and professional behaviour can never be conditioned by improper benefits of economic or other nature. Full disclosure is required, whenever conflicts of interest may impinge upon the diagnostic or therapeutic prescriptions or the possible relationships with industries and institutions [1].

It is also worth observing that a physician is very often an employee. The employer's and the patient's interest may not coincide and this can generate a conflict of loyalties. The distinction between external and internal goods, proposed by the philosopher Alasdair MacIntyre, helps clarify this ethical challenge. By *external goods*, we understand those goods, such as money, power and fame, that can be achieved as a result of the practice, but are not a component of its standard of excellence and tend to be enjoyed exactly as a *private* property. *Internal goods*, on the contrary, correspond to the very fundamental goals and essence of the practice and tend to be a good for all those, who are involved in it [2].

In the present “market society”, physicians are frequently faced with possible conflicts of interest in their prescriptions, but paediatricians are particularly vulnerable when they must decide how their patients should be fed. In the case of breast-feeding, the standard on which the internal good of excellence relies has been already set in the WHO's International Code of 1981: «Health workers should encourage and protect breast-feeding» and «no financial or material inducements» should be offered «to health workers or members of their families» nor accepted by them to promote the use of other products beyond their real need [3].
